# Beam Drift Mitigation and Wide-Range Measurement in a Miniaturized Ultrasonic Gas Flowmeter

**DOI:** 10.3390/mi17020254

**Published:** 2026-02-16

**Authors:** Shanfeng Hou, Xueying Xiu, Chengguang Liu, Haochen Lyu, Songsong Zhang

**Affiliations:** 1 School of Microelectronics, Shanghai University, Shanghai 201800, China; sf_hou@shu.edu.cn (S.H.); liuchengguang@shu.edu.cn (C.L.); 2 Melon Technologies Inc., Shanghai 201899, China; xueying.xiu@chiponsensing.com (X.X.); haochen.lv@chiponsensing.com (H.L.); 3 Department of Materials Science and Engineering, Rutgers University, Piscataway, NJ 08854, USA

**Keywords:** beam drift, ultrasonic gas flowmeters, piezoelectric micromachined ultrasonic transducer, half-power beamwidth

## Abstract

To mitigate acoustic beam drift, which degrades the signal-to-noise ratio (SNR) and limits the measurement range in ultrasonic gas flowmeters (USFMs), we present a miniaturized transit-time USFM that integrates a single piezoelectric micromachined ultrasonic transducer (PMUT) with a non-axisymmetric conical cavity. This design increases acoustic transmission gain and produces anisotropic directivity across orthogonal radiation planes, thereby broadening acoustic coverage along the flow direction and reducing beam steering. With an optimized cavity angle combination of (50°, 70°), the system achieves a 7.4 dB transmission gain and a half-power beamwidth (HPBW) of 29.1°. Experimental validation demonstrates a sound pressure attenuation of only 0.72 dB at 18.74 m/s. Within the 0.06–12 m^3^/h flow range, the USFM exhibits indication errors of ±2% (<1 m^3^/h) and ±1.5% (≥1 m^3^/h), with repeatability below 0.5%. The performance meets the Class 1.5 accuracy standard specified in CJ/T 477-2015, offering an innovative solution for wide-range miniaturized gas flow measurement.

## 1. Introduction

Flow measurement plays a pivotal role in automated inspection systems, and numerous technologies have been developed to enhance measurement accuracy across a wide range of applications [1,2]. Currently, diaphragm gas meters remain the most widely deployed devices for natural gas flow measurement in residential and commercial scenarios [3]. However, their inherent limitations, including limited-service life, relatively low measurement accuracy, and susceptibility to gas leakage [4], increasingly fail to meet the growing demand for high-precision and long-term stable metering.

Among these, ultrasonic flow measurement offers several distinct advantages, including a non-intrusive configuration, stable operational performance, minimal pressure loss, and a broad dynamic range [5,6]. These advantages make ultrasonic technology particularly attractive for next-generation industrial and domestic natural gas metering, as well as smart gas meter applications requiring compact integration and long-term reliability. In particular, ultrasonic flow meters (USFMs) based on the transit-time method demonstrate significant benefits, such as compatibility with various pipe diameters and fluid types, making them highly adaptable for diverse operational environments [7,8]. In transit-time-based USFMs, paired ultrasonic transducers alternately transmit and receive ultrasonic waves [8]. By comparing the time of flight for pulses sent with and against the flow, the fluid velocity can be determined [9].

However, under high-velocity gas flow conditions, the acoustic beam will be deflected due to flow velocity variations, which reduces the beam’s effective coverage area. This phenomenon causes significant signal-to-noise ratio (SNR) attenuation of the received signal and ultimately limits the measurement range of USFMs [10]. Current research has proposed multiple technical approaches to extend flow measurement ranges, yet it faces multi-level challenges. In signal processing, algorithm optimizations for SNR enhancement enable range extension [11,12,13] but increase computational complexity, hardware costs, and compromise real-time performance in complex environments. Acoustic compensation strategies encompass mechanical and electronic solutions: Mechanical approaches employ rotating transducers to establish tilted acoustic paths [14] or dynamically adjust receiver positions [15] for optimized wave reception efficiency, while phased array systems integrated with 3D-printed waveguide technology achieve non-mechanical beam steering compensation through phase manipulation [16,17,18]. Conventional USFMs utilizing bulk piezoelectric transducers (typically lead zirconate titanate, PZT) exhibit inherent limitations; their embedded cylindrical cavity structures disrupt flow-field symmetry, inducing unsteady vortex phenomena that distort velocity profiles and degrade measurement accuracy [19]. Additional constraints include poor component consistency and bulky dimensions, which not only impair beam-focusing capabilities in phased array systems but also necessitate enlarged pipe diameters, thereby restricting the miniaturization design requirements.

Fortunately, micromechanical ultrasonic transducers (MUTs) based on microelectromechanical systems (MEMS) offer clear advantages in miniaturization and system integration [20,21]. The MUT family comprises capacitive micromachined ultrasonic transducers (CMUTs) and piezoelectric micromachined ultrasonic transducers (PMUTs) [22,23]. Due to their low power consumption, linear response, and absence of bias voltage [24,25,26], PMUTs have gained increasing attention as acoustic transceivers in various applications [27,28,29,30,31]. Currently, researchers have used PMUTs for gas flow velocity measurements [32,33,34,35], yet practical measurements remain constrained by acoustic beam drift effects. Studies indicate that the final steering angle of the acoustic beam depends only on the average velocity of the fluid [36]. For a given volumetric flow rate, a smaller pipe diameter corresponds to a smaller cross-sectional area and therefore a higher average flow velocity, resulting in increased beam deflection. Consequently, ultrasonic flowmeters with smaller pipe diameters are more susceptible to beam drift under high flow conditions, making accurate measurement at high flow rates more challenging.

As beam drift increases, the received signal amplitude gradually decreases, with the extent of amplitude reduction depending on the main-lobe width [37]. From a practical engineering perspective, the −3 dB level (i.e., the half-power point) is commonly used as a reference to indicate a pronounced reduction in acoustic coupling. In this study, this level is adopted as an application-oriented guideline to characterize more significant beam drift effects.

In addition, emerging trends such as hydrogen blending in natural gas networks introduce further challenges for ultrasonic flow measurement. Changes in gas composition alter acoustic properties, including density, speed of sound, and attenuation, placing higher demands on the robustness of ultrasonic flowmeters against flow-induced and acoustic uncertainties.

This paper introduces a miniaturized transit-time USFM that combines a single PMUT with a non-axisymmetric conical cavity. The non-axisymmetric geometry increases acoustic gain in the desired direction and creates differentiated beamwidths in orthogonal planes, which together broaden coverage along the flow axis and mitigate beam drift. We present a transit-time USFM based on PMUT principles, detailing the rationale, design, and experimental validation of a non-axisymmetric cavity. The optimized (50°, 70°) cavity achieves a 7.4 dB gain and 29.1° HPBW, with only 0.72 dB attenuation at 18.74 m/s. The meter demonstrates ±2% (low flow) and ±1.5% (high flow) indication errors across 0.06–12 m^3^/h and repeatability below 0.5%, meeting CJ/T 477-2015 Class 1.5.

## 2. Transit-Time USFM Based on PMUT

### 2.1. Measurement Method

**Figure 1:**
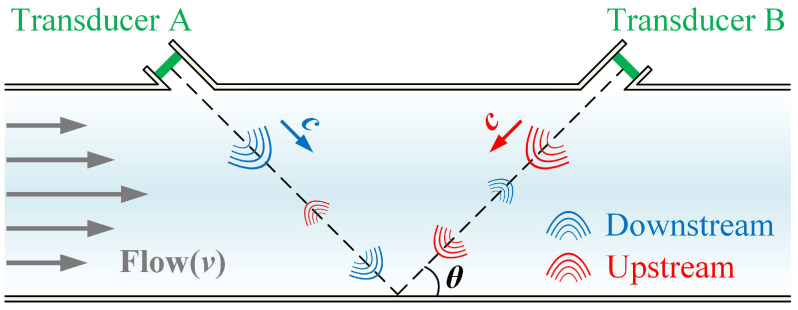
Schematic diagram of gas flow measurement principle of V-channel. The dashed lines indicate the ultrasonic propagation path between the two transducers.

The principle of the transit-time USFM is schematically shown in Figure 1. The measurement system uses a pair of identical piezoelectric transducers (Transducer A and B), installed at a specific angle of inclination on a pipe [38]. During operation, Transducer A first transmits ultrasonic pulses along the flow direction (downstream), and the corresponding time of flight (ToF) is recorded as *T_down_* (s). Subsequently, Transducer B emits pulses in the opposite direction (upstream), with the ToF denoted as *T_up_* (s). The basic principles are as follows:

(1) $$T_{down}=\frac{L}{c+\nu cos\theta },$$

(2) $$T_{up}=\frac{L}{c-\nu cos\theta }.$$

The axial flow velocity *v* (m/s) is obtained from their difference:

(3) $$\nu =\frac{L}{2cos\theta }\frac{\Delta T}{T_{up}T_{down}},\;\Delta T=T_{up}\;-\;T_{down}.$$

The flow rate *q* (m^3^/h) follows as

(4) $$q=k_{c}Sv=\frac{k_{c}SL\Delta T}{2cos\theta T_{up}T_{down}},$$

where *c* is sound speed (m/s), *L* is effective sound path (m), *θ* denotes the angle formed by the sound path and the pipe axis (°), Δ*T* is the difference time of flight (DToF) (s), *S* is the pipe cross-sectional area (m^2^) and *k_c_* is the calibration coefficient. Here, *k_c_* is used to correct for non-uniformity in the flow velocity distribution and installation effects and is usually determined through experimental calibration [39].

### 2.2. PMUT Fabrication and Characterization

**Figure 2:**
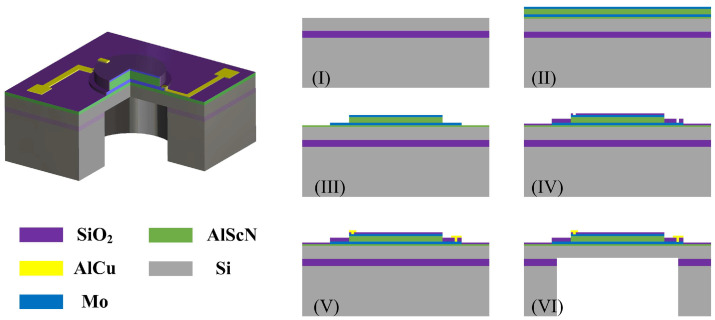
The 3D structure and fabrication of the PMUT. (I)–(VI) illustrate the main fabrication steps described in the text.

Figure 2 shows the three-dimensional (3D) structure of the PMUT. From bottom to top, the device consists of a 4 μm thick silicon substrate, a 0.05 μm AlScN seed layer, a 0.2 μm Mo bottom electrode, a 1 μm AlScN piezoelectric layer, and a 0.1 μm Mo top electrode. Compared with pure AlN, the AlScN film exhibits a higher piezoelectric coefficient and was deposited by reactive magnetron sputtering using a target with a Sc concentration of 20%. The silicon substrate is selectively etched by a backside anisotropic etching process, resulting in a circular diaphragm structure with an air resonance cavity measuring 600 μm in diameter and 400 μm in depth. The device achieves electromechanical energy conversion through the bidirectional piezoelectric effect of the AlScN thin film [40]. The PMUT fabrication process flow is as follows: (I) Preparation of a customized single-side polished silicon-on-insulator (SOI) wafer. (II) Sputtering and sequential deposition of AlScN/Mo/AlScN/Mo multilayer composite structures on SOI wafers [41]. (III) Patterning of the bottom Mo electrode, AlScN layer, and top Mo electrode via plasma etching. (IV) An oxide layer is deposited to form an isolation layer, and the upper and lower electrodes are etched via holes. (V) After etching, completion of PMUT interconnections occurs through metallization processes. (VI) Membrane release via deep reactive ion etching (DRIE) from the SOI wafer backside.

**Figure 3:**
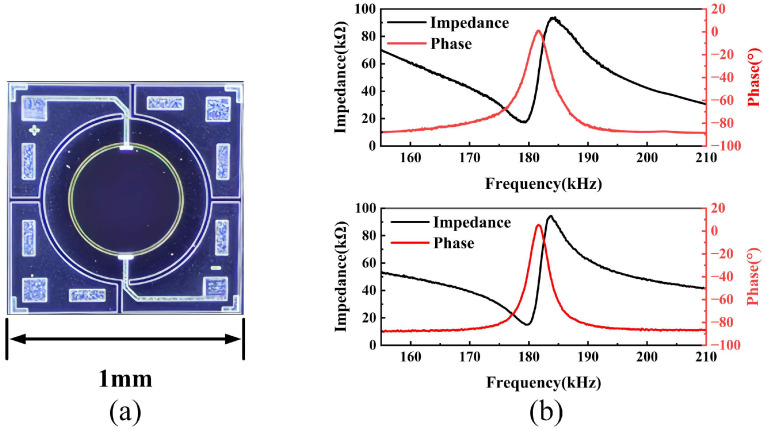
(**a**) The optical photo of the PMUT. (**b**) The measured impedance response of the PMUTs.

The resonant frequency of the transducer in USFMs is affected by the acoustic and thermodynamic properties of the working gas. Acoustic attenuation remains relatively low at frequencies below approximately 150 kHz, while it increases rapidly at frequencies above about 500 kHz due to gas absorption, which significantly degrades effective signal transmission [42]. On the other hand, higher operating frequencies generally provide improved acoustic directivity, which is beneficial for flow measurement in confined pipe geometries. Therefore, to ensure efficient signal propagation, we selected a frequency around 180 kHz. The PMUT optical photo is shown in Figure 3a. The PMUT electrical characteristics were measured using an impedance analyzer (E4990A, Keysight Technologies, Santa Rosa, CA, USA). As depicted in Figure 3b, the two PMUTs used in the flow measurement experiments were fabricated on the same wafer using identical processes and share the same structural and material parameters. Their measured resonant frequencies are highly consistent, centered at approximately 181.6 ± 0.1 kHz, which is in accordance with the design.

**Figure 4:**
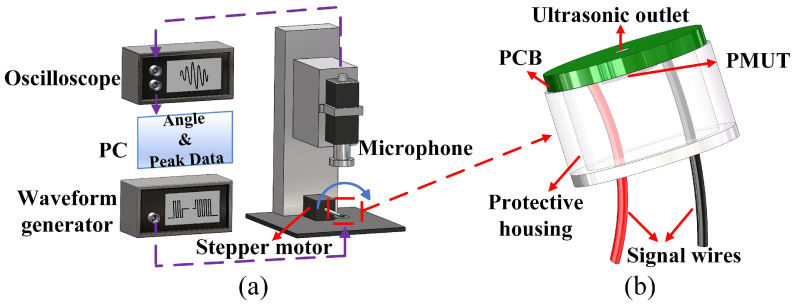
(**a**) Schematic diagram of the acoustic test platform. (**b**) Package of the PMUT.

**Figure 5:**
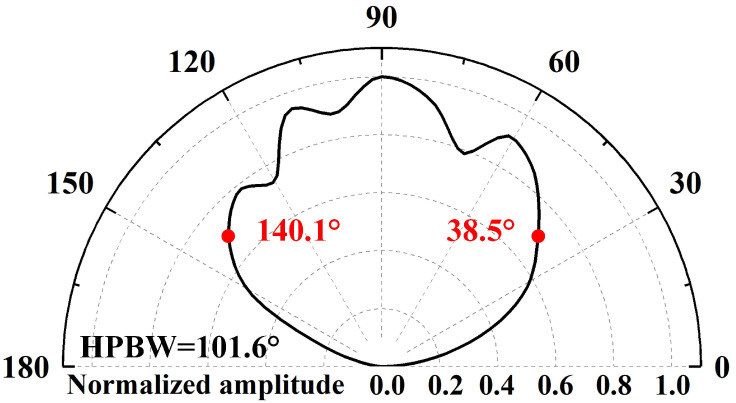
Experimental results of directivity.

The directivity of ultrasonic transducers, defined as the variation in transmission response amplitude with azimuthal angle, is a critical performance parameter. As shown in Figure 4a, our experimental setup employed the following workflow: First, we mounted the PMUT on the central rotating shaft of a stepper motor and drove it using a burst signal (181.6 kHz, 5 Vpp, 20 cycles) generated by a waveform generator (Keysight Technologies, 33600A Series). Subsequently, a high-sensitivity capacitive microphone (CM16/CMPA-5V, Avisoft Bioacoustics, Glienicke/Nordbahn, Germany) captured acoustic field signals, which an oscilloscope (Keysight Technologies, DSOX2024A) transmitted to a PC in real time. This study employs a resonant cavity to emit ultrasonic waves, thereby increasing the transmitted acoustic pressure of a single PMUT element [43]. As shown in Figure 4b, the PMUT was bonded to a printed circuit board (PCB) using gold wire interconnects, with signals routed to external systems via the PCB. The front surface of the PMUT was encapsulated with a protective housing, enabling ultrasonic waves from the resonance cavity to propagate through the hole in the PCB. During testing, the stepper motor rotated from 0° to 180° in 1.8° increments, capturing acoustic pressure amplitude data at 101 spatial sampling points to reconstruct the full polar radiation pattern. To characterize the PMUT’s directivity, we used the half-power beamwidth (HPBW) (°) as the quantitative metric [44]. As shown in Figure 5, the −3 dB attenuation point corresponds to an azimuth angle ranging from 38.5° to 140.1°, and the HPBW is calculated as 101.6°. In addition to the normalized directivity patterns, an absolute acoustic reference was obtained by measuring the acoustic pressure generated by the PMUT under the same excitation conditions. At a distance of 1 cm above the device, the effective transmit sensitivity was approximately 0.43 Pa/V, which is provided as an engineering reference for the acoustic output level.

## 3. Design and Experiments of the Non-Axisymmetric Cavity

### 3.1. Mathematical Analysis

**Figure 6:**
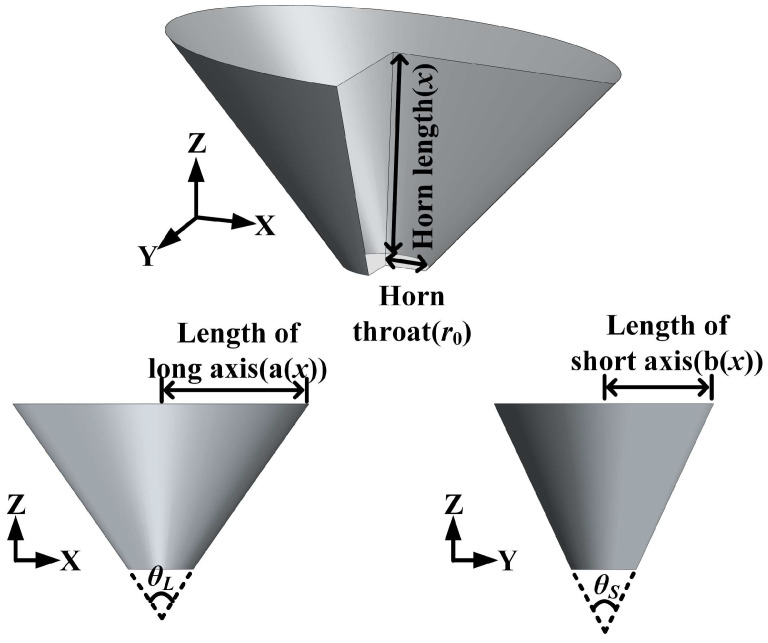
Schematic diagram of conical horn.

As shown in Figure 6, the conical transducer cavity is designed as a structure with a circular throat and an elliptical opening, which can be thought of as analogous to a finite-length acoustic horn. Assuming the cross-sectional area varies along its axial length *x* (m), the cross-sectional area is expressed as *S* = *S*(*x*) (m^2^). Considering the wavefront evolution governed by the law of transversality, the sound pressure *p* (Pa) satisfies the following wave equation [45]:

(5) $$\frac{\partial^{2}p}{\partial x^{2}}+\left(\frac{\partial lnS}{\partial x}\right)\frac{\partial p}{\partial x}=\frac{1}{c^{2}}\frac{\partial^{2}p}{\partial t^{2}}.$$

Assuming a time-harmonic solution of the form $p=p(x)e^{j\omega t}$, (6) reduces to an ordinary differential equation in terms of x:

(6) $$\frac{d^{2}p\left(x\right)}{dx^{2}}+\frac{S^{\prime }}{S}\frac{dp\left(x\right)}{dx}+k^{2}p\left(x\right)=0,$$

where *k* = *ω*/*c* is the acoustic wavenumber. *S* can be expressed as follows:

(7) $$S\left(x\right)=\pi \cdot a\left(x\right)\cdot b\left(x\right)=\pi \cdot \left(r_{0}+x\tan\left(\frac{\theta_{L}}{2}\right)\right)\left(r_{0}+x\tan\left(\frac{\theta_{S}}{2}\right)\right),$$

where *a*(*x*) and *b*(*x*) denote the major and minor axis lengths (m), while *θ_L_* and *θ_S_* denote the horn cone angle on the face where the long and short axes are located, respectively (°).

In the special case where *θ* = *θ_L_* = *θ_S_*, the conical horn becomes fully axisymmetric, and its solution reduces to

(8) $$p=\frac{1}{r_{0}+x\tan\frac{\theta }{2}}\left[C_{1}e^{i\omega \left(t-\frac{x}{c}\right)}+C_{2}e^{i\omega \left(t+\frac{x}{c}\right)}\right],$$

where *C*_1_ and *C*_2_ are complex constants determined by the boundary conditions. When *θ_L_* ≠ *θ_S_*, the conical horn is no longer axisymmetric. Through mathematical analysis, the equations for the non-axisymmetric conical horn have no universal analytical solution. It will be analyzed using asymptotic approximation below.

#### 3.1.1. Short-Range Approximation

Let *m = tan(θ_L_/2)* and *n = tan(θ_S_/2)*. When *x* ≪ *r*_0_/*m*, and *x* ≪ *r*_0_/*n*, we approximate the cross-sectional area as

(9) $$S\left(x\right)\approx \pi r_{0}^{2}\left(1+\frac{\left(m+n\right)}{r_{0}}x\right),$$

(10) $$\frac{S^{\prime }\left(x\right)}{S\left(x\right)}\approx \frac{\pi r_{0}\left(m+n\right)}{\pi r_{0}^{2}\left(1+\frac{\left(m+n\right)}{r_{0}}x\right)}\approx \frac{m+n}{r_{0}}\left(1-\frac{\left(m+n\right)}{r_{0}}x\right).$$

Assuming that the solution is in a traveling waveform,

(11) $$p\left(x\right)=A\left(x\right)e^{ikx},$$

where the amplitude *A(x)* varies slowly (satisfying |*A*″| ≪ *k*|*A*′|). We neglect higher-order terms *A*″ and impose *S*/*S*′ ≪ *k*. This simplification yields the approximate solution

(12) $$A\left(x\right)\approx \frac{C_{1}}{\sqrt{1+\frac{\left(m+n\right)}{r_{0}}x}}.$$

And consequently,

(13) $$p\approx \frac{C_{1}}{\sqrt{1+\frac{\left(m+n\right)}{r_{0}}x}}e^{i\omega \left(t+\frac{x}{c}\right)}.$$

#### 3.1.2. Long-Range Approximation

When *x* ≪ *r*_0_/*m*, and *x* ≪ *r*_0_/*n*, we approximate the cross-sectional area as

(14) $$S\left(x\right)\approx \pi mnx^{2},$$

(15) $$\frac{S^{\prime }\left(x\right)}{S\left(x\right)}\approx \frac{2}{x}.$$

Substituting (15) into (6) gives

(16) $$p\approx \frac{1}{x}\left(C_{1}e^{i\omega \left(t-\frac{x}{c}\right)}+C_{2}e^{i\omega \left(t+\frac{x}{c}\right)}\right).$$

The analysis shows that the sound transmission characteristics of the conical horn depend on the multi-parameter synergistic control mechanism of the throat radius, cone angle parameter, frequency and horn height. By optimizing the above geometrical and acoustic parameters, the acoustic beam focusing characteristics and the dynamic response characteristics of energy attenuation can be regulated.

### 3.2. Finite Element Analysis and Experimental Results

**Figure 7:**
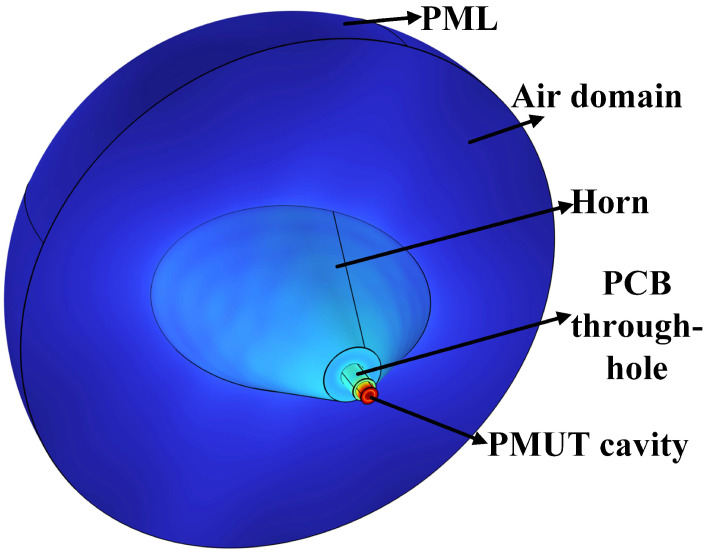
Three-dimensional simulation model.

As shown in Figure 7, a 3D simulation model was established based on Finite Element Method (FEM) software (COMSOL Multiphysics 6.2, COMSOL AB, Stockholm, Sweden). The model adopts a layered modeling method, which contains the PMUT resonant cavity, PCB through-hole, horn structure, air domain, and Perfectly Matched Layer (PML) boundary conditions in turn. The diameter and vertical dimension of the PMUT resonant cavity are set to 0.6 mm and 0.4 mm, respectively; the PCB through-hole is parameterized to have a height of 1 mm and a diameter of 0.8 mm. To avoid obstruction to the acoustic wave propagation, the throat radius *r*_0_ was set to 1 mm. The horn height *x* was set to 5.1 mm to match the cylindrical transducer cavity height used in a commercial bulk PZT-based USFM (UG-YC4+, Zhejiang Weixing Intelligent Meter Co., Ltd., Hangzhou, Zhejiang, China), enabling engineering-level comparison. An acoustic excitation source with a frequency of 181.6 kHz was set in the PMUT resonant cavity, and the size and shape of the elliptical exit surface were regulated by controlling the variables and parametrically scanning the horn opening angles *θ_L_* and *θ_S_*. Eight angular gradients are constructed in the range of 10–80° in 10° steps to form 36 sets of (C_8_^2^ + 8) parameter combinations for frequency domain simulations.

**Figure 8:**
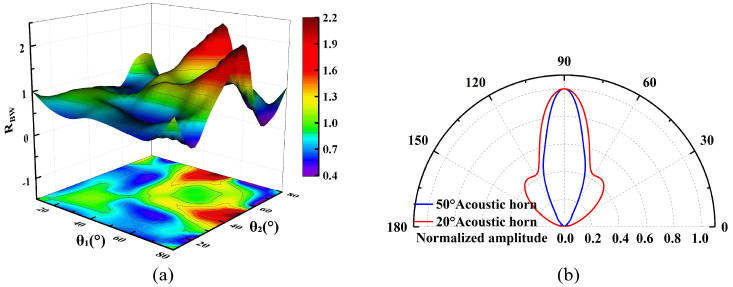
(**a**) Simulated *R_BW_* variation for different combinations of cone angles. (**b**) Normalized acoustic directivity for typical combination (*θ_S_*, *θ_L_*) = (20°, 50°).

In radiated sound field characterization, the long-axis component (HPBW-L) and the short-axis component (HPBW-S) of the HPBW are defined as the angular span corresponding to a 3 dB drop in sound pressure level from the main lobe peak in the radiation plane containing the long and short axes of the elliptical opening, respectively. The ratio of the two (*R_BW_* = HPBW-L/HPBW-S) quantitatively characterizes the focusing variability of the acoustic field energy in the orthogonal direction. Figure 8a demonstrates the variation of *R_BW_* for different combinations of cone angles (*θ_S_* = min (*θ*_1_, *θ*_2_), *θ_L_* = max (*θ*_1_, *θ*_2_)). When *θ_S_* = *θ_L_*, the conical cavity exhibits axisymmetric characteristics, at which HPBW-L and HPBW-S are equal (*R_BW_* = 1) and *R_BW_* varies parabolically with the increase of *θ*, reaching a minimum value of 19.07° at *θ* = 50°, which indicates that the axisymmetric structure can achieve optimal beam focusing. However, when *θ_S_* ≠ *θ_L_*, *R_BW_* deviates from the benchmark value (*R_BW_* ≠ 1): when *R_BW_* > 1, the beam broadening effect in the long-axis direction dominates, and the acoustic field exhibits anisotropic diffusion; whereas when *R_BW_* < 1, it exhibits an inverse modulation pattern of beam broadening in the short-axis direction and beam contraction in the long-axis direction. It is worth noting that in the short-axis radiation plane with *θ_S_* = 10° or 20° (e.g., the typical combination of (*θ_S_*, *θ_L_*) = (20°, 50°)), significant side lobes appear around the main lobe (Figure 8b). The elevated energy share of the side lobes will induce clutter interference, leading to a deterioration of the SNR and an increase in the measurement error. From an engineering perspective, excessive sidelobe levels may introduce interference and reduce effective main-beam energy. In this study, sidelobe suppression was treated as a practical design consideration to balance acoustic directivity and ToF stability, rather than pursuing maximum beam narrowing. The simulation results show that the non-axisymmetric conical cavity can realize differentiated acoustic field directivity regulation in different radiation planes by destroying the axial symmetry of the acoustic impedance distribution, which provides a new direction for directional acoustic energy transmission and beam shaping.

**Figure 9:**
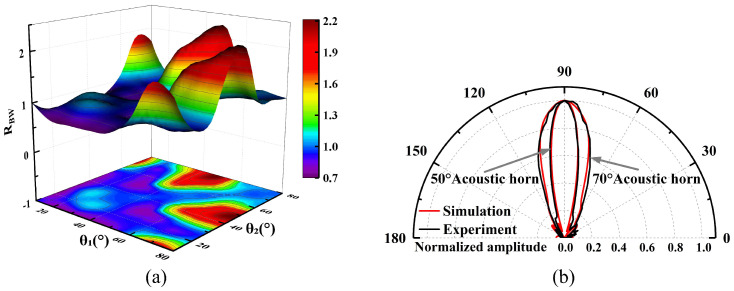
(**a**) Experimental *R_BW_* variation for different combinations of cone angles. (**b**) Normalized acoustic directivity for target cavity combination (*θ_S_*, *θ_L_*) = (50°, 70°).

The 36 conical cavity structures were fabricated using polylactic acid (PLA) material via 3D printing for experimental validation, and the results are shown in Figure 9a. The measured acoustic beam characteristics are basically in agreement with the simulation trend, where the small deviations originate from the manufacturing and testing errors. In USFM design, to optimize the performance of directional acoustic wave transmission, two design considerations are based on the following: first, in order to enhance the signal reception efficiency in the flow direction and, at the same time, to constrain the lateral opening to reduce the interference of multipath reflections, the lateral direction is designed to be the short-axis direction; and second, to achieve the acoustic wave coverage extension in the flow direction, *R_BW_* should be maintained at >1. To synergistically realize the signal gain and beam spreading, this study constructs a two-stage screening process by taking the measured HPBW (18.8°) of the 50° axisymmetric conical cavity as the reference threshold. First, the conical cavities satisfying the conditions of HPBW-S ≤ 18.8° ≤ HPBW-L are screened out, as shown in Table 1; second, the optimal configuration corresponding to the maximum signal gain is selected from them. The gain *g* (dB) of amplitude is defined as *g* = 20*lg(V_θ_/V_0_)*, where *V_θ_* is the amplitude of the transmitted signal with the cavity (V), and *V_0_* is the transmitted signal amplitude without the cavity (V). As evidenced by the data in Table 1, beam broadening is achieved at the cost of partial signal gain reduction compared to the 50° axisymmetric conical cavity, further verifying that signal gain and beam broadening are mutually constrained. As shown in Figure 9b, the configuration with the angle combination of (*θ_S_*, *θ_L_*) = (50°, 70°) was selected as the target cavity. The experimentally measured acoustic beam directivity data are in good agreement with the simulation results. Although the gain of this target configuration is reduced by 0.91 dB compared with the 50° axisymmetric reference configuration, its beamwidth is significantly increased by 63.3% in the long-axis direction. This result confirms the engineering feasibility of asymmetric acoustic impedance matching mechanisms, enabling directional beam coverage expansion in the major axis while maintaining an acceptable SNR.

### 3.3. Experimental Screening and Target Selection

**Figure 10:**
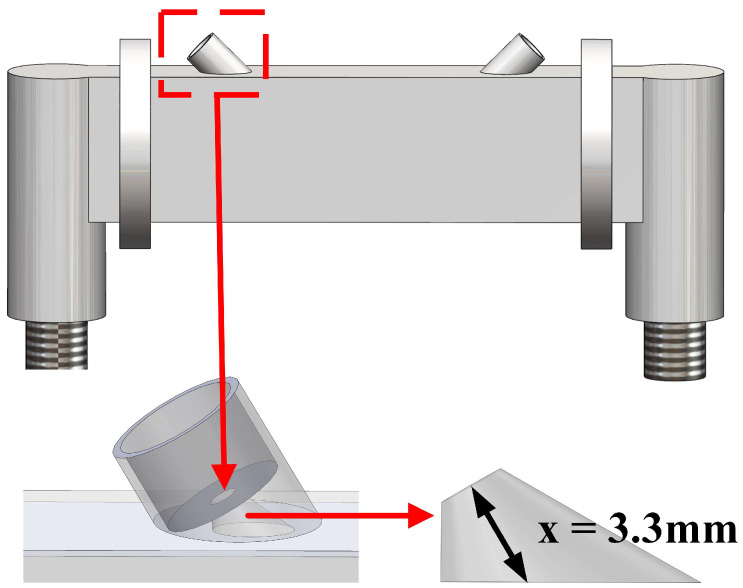
Model of conical transducer cavity pipe. The red dashed box indicates the enlarged region shown below, and the red arrow denotes the corresponding position.

Figure 10 shows that tilting the transducer reduces the center-to-wall distance from 5.1 mm to 3.3 mm. This inclined configuration departs from the standard conical horn, and its acoustic field behavior within the pipe system requires further experimental verification. In particular, although the inclination of the transducer cavity essentially breaks the symmetry of the structure, the terminology system of “axisymmetric” and “non-axisymmetric” is retained in the subsequent discussion to distinguish the features for the sake of convenience of description. The acoustic module designed in this study was mounted on the acoustic test platform shown in Figure 4a to analyze the acoustic directivity in the plane of the flow direction in the pipe.

**Figure 11:**
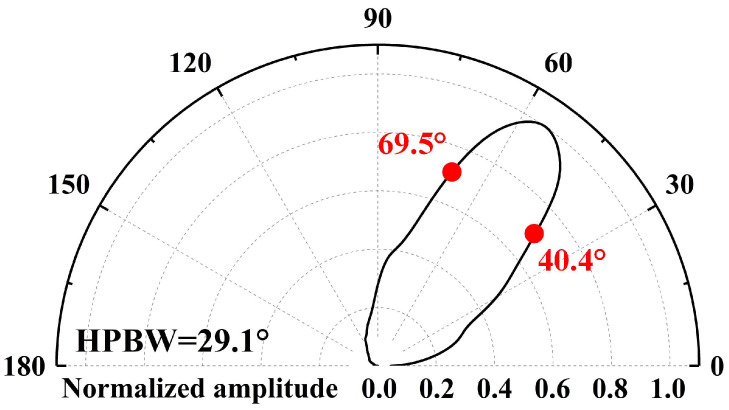
Acoustic directivity in the plane of flow direction for (50°, 70°) combined angles.

To verify that the non-axisymmetric conical cavity maintains anisotropic characteristics in acoustic beam modulation when tilted, axisymmetric conical cavities were also tested for comparative analysis. Table 2 presents a comprehensive comparison of the experimental data. Analysis reveals that compared with axisymmetric cavities having the same single-angle value, the non-axisymmetric cavities exhibit both beam broadening and high gain characteristics. For example, the axisymmetric cavity has the narrowest beam and the highest gain when the cone angle is 50°, but when the cone angle is increased to 70°, the gain decreases severely despite the beam broadening. In contrast, the non-axisymmetric cavity is only 0.4 dB lower than the 50° axisymmetric cavity at the (50°, 70°) combined angles, while the beam broadens by 52.4%. Notably, although both types of cavities are designed with a 60° inclination angle, their acoustic peaks deviate from the preset angle and show characteristics of being less than 60°, which correlates with their intrinsic structural asymmetry. As shown in Figure 11, the (50°, 70°) non-axisymmetric cavity reaches maximum acoustic intensity at a 55° azimuth. Based on this phenomenon, the received signal amplitude is maximized by appropriately increasing the transducer spacing while keeping the transducer transmitting at a 60° inclination angle.

## 4. Experimental Results

### 4.1. Experimental System

**Figure 12:**
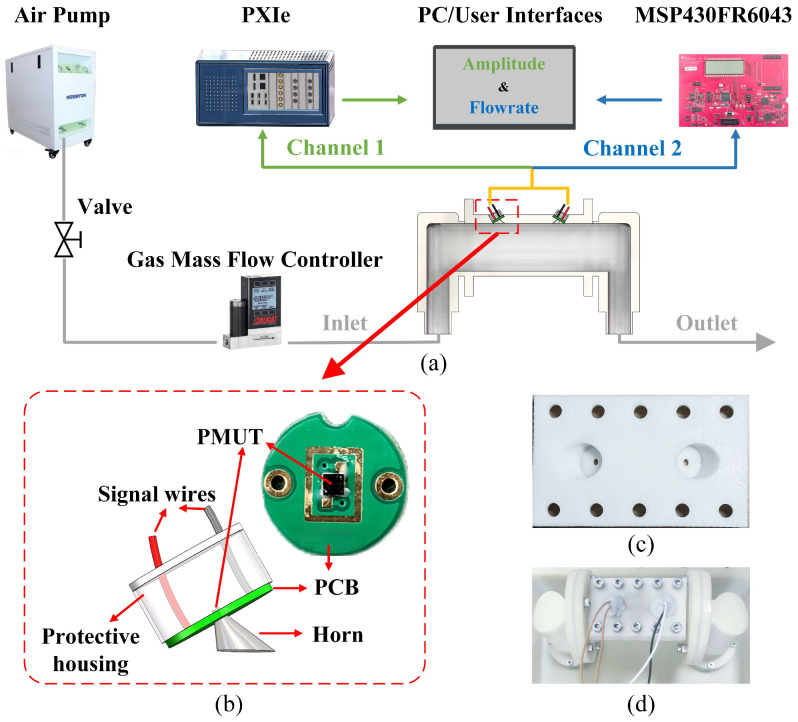
Experimental configuration and assembly details of the proposed ultrasonic gas flowmeter. (**a**) Schematic diagram of the experimental system. (**b**) CAD view and photograph of the PMUT module, illustrating the geometric relationship between the PMUT, PCB, and horn cavity. (**c**) Photograph of the fabricated pipe cover integrating the horn cavity and a predefined mounting recess for the PMUT module at a specified inclination angle. (**d**) Photograph of the assembled flowmeter prototype, showing the PMUT module fixed within the horn-related mounting structure using an adhesive material. The green and blue arrows represent Channel 1 and Channel 2 signal paths, respectively, while the red arrows and dashed boxes indicate enlarged structural regions.

**Figure 13:**
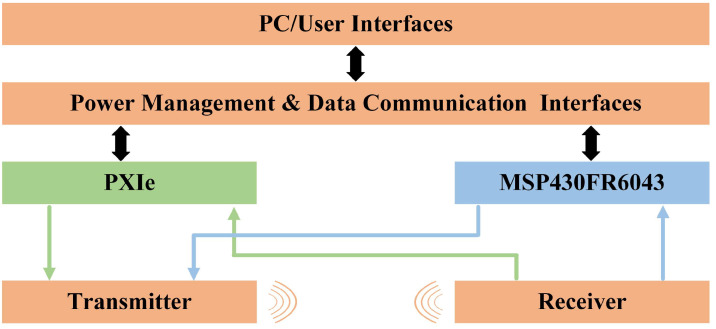
Block diagram of the USFM system. The green and blue arrows denote different signal transmission paths within the system. The wave icons represent ultrasonic signal propagation.

The experimental setup and system are built as shown in Figure 12. The experimental system consists of three parts: the pneumatic module, the flowmeter module, and the data acquisition system. The pneumatic module employs an air pump as the driving source. A commercial mass flow controller (MC Series, Alicat Scientific, Tucson, AZ, USA), factory-calibrated and traceable to the National Institute of Standards and Technology (NIST), was used as the reference instrument for flow calibration. The device was operated within its valid calibration period throughout all experiments. In the flowmeter module, which has a cross-section of 10.2 mm in width and 21.8 mm in height, the sensors adopt a V-shaped configuration to enhance detection sensitivity by extending the effective acoustic path length in a compact geometry [9]. In our setup, they were tilt-mounted on one side of the pipe, with their center axis forming a 60° angle with the axial direction of the pipe. To ensure a stable flow field, we integrated a rectifier plate into the measurement pipe [46]. The data acquisition system utilizes a modular instrumentation platform (National Instruments, Austin, TX, USA) working with the ultrasonic sensing microcontroller (MSP430FR6043, Texas Instruments, Dallas, TX, USA). As shown in Figure 13, the experimental procedure comprises two phases: acoustic beam drift verification and flow measurement performance validation. The system utilizes the PXIe module’s high-precision signal acquisition, real-time processing capabilities, and multistage anti-aliasing filtering technology to continuously monitor amplitude variations in raw received signals, thereby validating the beam stability of the PMUT integrated with the non-axisymmetric cavity. Subsequently, flow measurement performance is evaluated using the MSP430FR6043 microcontroller, which enables acquisition of ToF and DToF data. In our setup, the PMUT is connected to the MSP430FR6043, and bidirectional signal transmission is achieved using a 3.3 Vpp excitation voltage and time-division multiplexing (TDM). Data acquisition was performed with a graphical user interface (GUI); the recorded measurements were converted to flow rates using Equation (4).

### 4.2. Zero-Flow Stability

**Figure 14:**
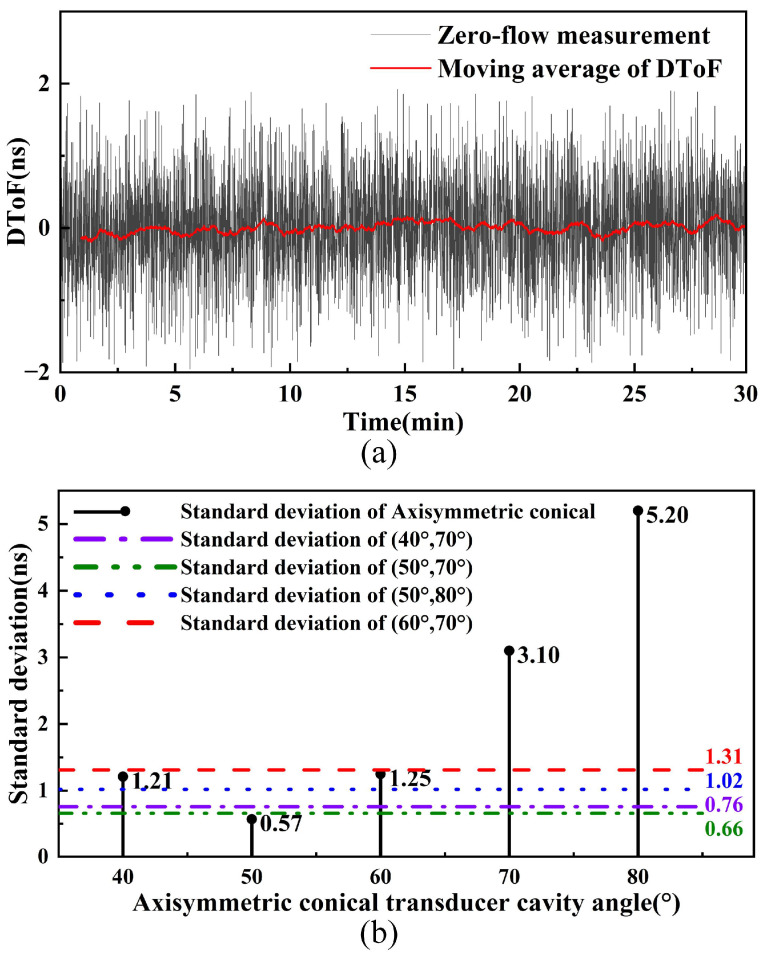
Zero-flow error measurement results. (**a**) (50°, 70°) horn zero-flow results. (**b**) Standard deviation of zero-flow for different cone angles.

To evaluate the effect of cavities with different angles on measurement accuracy, zero-flow experiments were performed at room temperature using transit-time USFM. Under zero-flow conditions, random errors of the measured DToF can reflect the SNR of the system. Each experiment lasted 30 min with a 0.5 s sampling interval to ensure adequate data density. Figure 14a demonstrates the DToF measurements of the (50°, 70°) conical cavity under zero-flow conditions. Experimental data demonstrate that individual DToF measurements exhibit fluctuations within ±2 ns, with a standard deviation of 0.66 ns, indicating excellent consistency between the two PMUT transducers. By setting a 1 min time window, the fluctuation amplitude of the moving average converges to within ±200 ps. Further analysis through Figure 14b, which compares measurement stability between the two cavity types at different cone angles, reveals that the axisymmetric cavity achieves its optimal standard deviation of 0.57 ns at a 50° cone angle, whereas the non-axisymmetric cavity attains peak performance at the combined angles (50°, 70°). Considering both beam broadening characteristics and acoustic intensity gain, the (50°, 70°) non-axisymmetric cavity is finally selected as the optimal design.

### 4.3. Acoustic Beam Drift Verification

To evaluate the beam stability of the non-axisymmetric conical cavity, the acoustic beam drift experiment was conducted using Channel 1 of the experimental system shown in Figure 12. The flow rate range of 2.5–15 m^3^/h was divided into six gradients at 2.5 m^3^/h intervals (2.5, 5.0, 7.5, 10.0, 12.5, 15.0 m^3^/h), with each gradient corresponding to distinct flow velocity conditions. The maximum flow rate of 15.0 m^3^/h corresponds to an average flow velocity of 18.74 m/s. The experiments were conducted in ascending order of flow values, and each target flow point was tested under zero-flow conditions to obtain the baseline amplitude data. After the target flow rate stabilized, the system obtained the mean amplitude of acoustic signals through continuous 1 min measurements and calculated the amplitude attenuation relative to the baseline state. In this study, a research architecture of comparative analysis between an experimental group and a double control group was used to investigate the mechanism of the influence of the cavity geometric configuration on the performance of the USFM. The experimental group configuration features a PMUT-based USFM with a non-axisymmetric conical cavity. The control group setup consisted of two configurations: Control Group 1 was based on a cylindrical cavity USFM with a commercial bulk piezoelectric ultrasound transducer (PSC-200k, Zhejiang Jiakang Electronics Co., Ltd., Jiaxing, Zhejiang, China), operating at 200 kHz with an HPBW of 13.2°, and Control Group 2 maintained the same PMUT as the experimental group but with a 50° axisymmetric conical cavity configuration.

**Figure 15:**
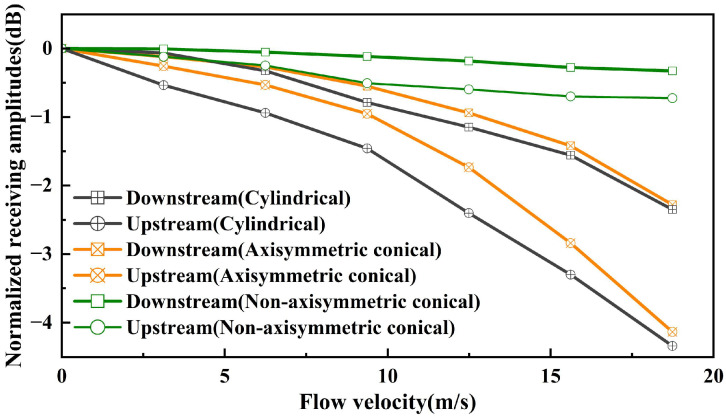
Normalized amplitude of the received signal along the downstream and upstream paths versus flow velocity.

Figure 15 shows the normalized amplitude (dB) variations of received signals along downstream and upstream propagation paths for three groups of USFMs across the 0–18.74 m/s flow velocity range. Experimental data reveal that as flow velocity increases, all transducers exhibit decreasing signal amplitudes in both flow directions, with upstream paths demonstrating significantly higher sensitivity to velocity variations compared to downstream paths. Further analysis demonstrates a strong negative correlation between beamwidth and velocity sensitivity: narrower beamwidths are susceptible to flow velocity. During upstream propagation, Control Group 1, which has the narrowest beamwidth, exhibits a maximum acoustic pressure level attenuation of 4.34 dB, whereas the experimental group, with a broader beamwidth, shows only 0.72 dB attenuation. Control Group 2, whose beamwidth lies between that of the experimental group and Control Group 1, displays a maximum signal attenuation of 4.13 dB, exhibiting intermediate characteristics corresponding to its beamwidth.

### 4.4. Flow Measurement Performance Validation

To systematically evaluate the measurement accuracy and repeatability characteristics of the developed USFM, this study performs flow calibration experiments using Channel 2 of the experimental system shown in Figure 12. According to the Chinese national standard for ultrasonic gas meters (CJ/T 477-2015) [47], air is permitted as a substitute test medium for performance evaluation; therefore, it was adopted in this study. Following the national standard, ten characteristic flow points are selected within the 0.06–12 m^3^/h range (*q_min_* = 0.06 m^3^/h, 3*q_min_*, 5*q_min_*, 10*q_min_*, *q_t_* = 1 m^3^/h, 0.2*q_max_*, 0.4*q_max_*, 0.7*q_max_*, *q_max_* = 10 m^3^/h, and 1.2*q_max_*). Measurements were carried out under constant temperature conditions. For each flow point, three independent calibration trials were performed. After flow stabilization, continuous 1 min measurements were taken, and the mean of the three measurements was used as the calibrated value. The calibration factor was determined as the ratio between the reference flow value and the calibrated value. Experimental data are analyzed through nonlinear regression using a second-order exponential decay function model (17). The fitted parameters yield *a* = 1.641, *b* = 0.0012, *c* = −1.632, and *d* = −11.6, with a coefficient of determination *R^2^* = 0.9922, confirming the model’s effectiveness in characterizing the experimental data distribution.

(17) $$y=ae^{bx}+ce^{dx}.$$

Following calibration, repeated measurements were performed at each flow point under controlled experimental conditions, using the same experimental group–dual control group comparative framework described in the previous section. The test sequence proceeded with three successive increasing-flow cycles followed by three successive decreasing-flow cycles. Measurement uncertainty was evaluated using a Type A statistical approach, in which the standard deviation of repeated measurement cycles was used to characterize measurement variability and repeatability under identical operating conditions. The flowmeter performance is quantitatively characterized by the indication error *E_ij_* (18) and the repeatability error *E_ri_* (19), where *q_ij_* denotes the *j*-th measured value at the *i*-th flow rate point, *Q* is the reference flow value (m^3^/h), and *E_i_* is the average indication error at the corresponding flow point [47].

(18) $$E_{ij}=\frac{q_{ij}-Q}{Q}\times 100\%,$$

(19) $$E_{ri}={\left[\frac{1}{6-1}\sum_{j=1}^{6}{\left(E_{i}-E_{ij}\right)}^{2}\right]}^{\frac{1}{2}}.$$

**Figure 16:**
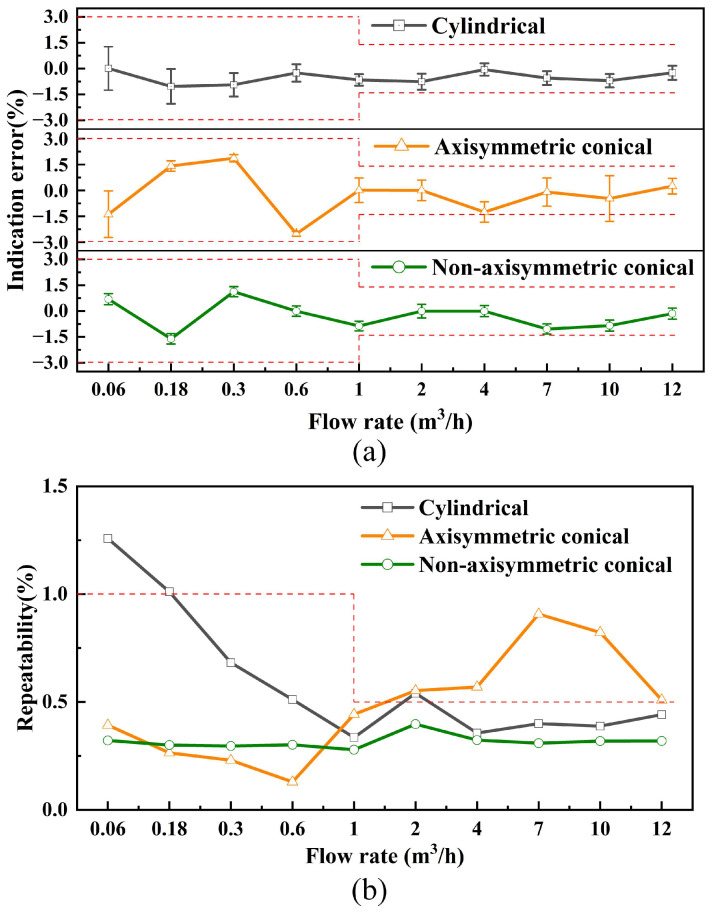
The red dashed lines represent the maximum permissible error and maximum repeatability specified in the Chinese national standard CJ/T 477-2015 for Class 1.5 gas meters. (**a**) Indication error and (**b**) repeatability error of the ultrasonic flowmeter as a function of flow rate. Each data point corresponds to the mean value of repeated measurements. Error bars represent the standard deviation of six measurement cycles, which is used as a Type A evaluation of measurement uncertainty. The indication error is calculated relative to the reference flow measured by a NIST-traceable mass flow controller.

The post-calibration indication error analysis shown in Figure 16a demonstrates significant performance advantages of the non-axisymmetric conical cavity USFM across different flow rate intervals. In flow rate ranges where *q* < *q_t_*, the measurement error remains within ±2.0%, which is better than that of the axisymmetric conical cavity USFM. For *q* ≥ *q_t_*, the non-axisymmetric configuration maintains an error limit of ±1.5%, whereas the axisymmetric conical cavity USFM exceeds the standard allowable range. Figure 16b reveals that the non-axisymmetric conical cavity USFM achieves full-scale repeatability errors below 0.5%, complying with the repeatability specifications for Class 1.5 accuracy instruments. Currently, the axisymmetric conical cavity USFM and the cylindrical cavity USFM cannot meet the same standard.

The USFM developed in this study demonstrates improved accuracy and repeatability, which is attributed to the acoustic field regulation introduced by the non-axisymmetric conical cavity. In ToF-based detection, beam drift caused by flow velocity results in variations in the effective acoustic propagation path and arrival angle, introducing ToF deviations and instability. The non-axisymmetric cavity alters the acoustic beam distribution by broadening the effective beamwidth in the flow direction and reducing lateral energy spread. This makes the received signal less sensitive to flow-induced angular deviations and keeps the dominant acoustic energy aligned with the designed propagation path, thereby reducing ToF fluctuations and improving indication accuracy. Additionally, the suppression of sidelobe energy and the concentration of acoustic energy improve the SNR at the receiver. This enhanced SNR stabilizes the ToF extraction process, which contributes to the improved repeatability observed in Figure 16b. Thus, the non-axisymmetric conical cavity enhances both indication accuracy and repeatability by stabilizing the acoustic propagation path and minimizing the impact of flow-induced beam drift in ToF-based measurements.

## 5. Discussion

The improved high-flow robustness of the proposed design originates from the anisotropic directivity of the non-axisymmetric conical cavity. By employing different cone angles along orthogonal axes, the cavity forms an elliptical radiation pattern that broadens the beam in the flow direction while constraining lateral spread. This geometric trade-off increases angular tolerance to flow-induced beam steering, reducing the sensitivity of the received amplitude to small angular deviations and thereby preserving the SNR over an extended measurement range. Consequently, a larger fraction of acoustic energy remains incident on the receiver under high-velocity flow conditions. Experimentally, this results in only 0.72 dB attenuation at 18.74 m/s, along with a more stable received waveform and reduced DToF jitter. These improvements enable reliable ToF extraction across a wider flow range without sacrificing practical acoustic gain.

From an engineering perspective, the non-axisymmetric cavity provides a compact and manufacturable solution to extend the operating range of PMUT-based ultrasonic flow meters, avoiding the complexity of transducer arrays or mechanical beam steering. However, this design involves inherent trade-offs. Certain cone-angle combinations can introduce sidelobes that degrade the SNR, while fabrication tolerances and assembly misalignment may shift the acoustic peak. Future work will therefore focus on geometry optimization under manufacturing constraints, as well as adaptive cavity designs to further enhance robustness.

In this study, the experimental evaluation was conducted in air, in accordance with the standardized testing conditions described in Section 4.4. Compared with air, natural gas exhibits different acoustic and thermodynamic properties, including density, speed of sound, and acoustic attenuation. In ToF-based ultrasonic flowmeters, these differences primarily affect the absolute ToF value and signal attenuation, which can be compensated for through gas-specific calibration parameters. Importantly, the core improvement proposed in this work—the suppression of flow-induced beam drift through a non-axisymmetric conical cavity—is achieved via geometric acoustic field regulation rather than gas-dependent material properties. Therefore, the proposed improvement mechanism is not inherently dependent on a specific gas medium, although quantitative performance may vary and require appropriate gas-specific calibration.

It should be noted that experimental validation under actual natural gas and hydrogen-enriched gas conditions has not yet been carried out in the present study. As a result, the reported performance metrics are limited to controlled laboratory conditions using air as the test medium, as permitted by the Chinese National Standard CJ/T 477-2015. This defines the current scope of the study and highlights the need for extended validation under practical gas compositions in future studies.

## 6. Conclusions

In this paper, we have presented a miniaturized transit-time ultrasonic gas flowmeter that integrates a single PMUT with a non-axisymmetric conical cavity to mitigate acoustic beam drift and extend the measurement range. Numerical analysis and experimental screening identified the (50°, 70°) cavity as an optimal compromise between beam broadening and amplitude gain. The system achieves a transmit signal gain of 7.4 dB while maintaining a wider HPBW of 29.1°. Experimental results show that under a high flow velocity of 18.74 m/s, the USFM exhibits only 0.72 dB of acoustic pressure attenuation, effectively mitigating beam drift effects. Within the 0.06–12 m^3^/h flow range, the USFM exhibits indication errors of ±2% in the low-flow regime (<1 m^3^/h) and ±1.5% in the high-flow regime (≥1 m^3^/h), with repeatability below 0.5%. This design complies with Class 1.5 accuracy requirements of the Chinese National Standard CJ/T 477-2015, providing an innovative solution for wide-range measurement in miniaturized gas flow detection systems.

## Figures and Tables

**Table 1:** Conical cavity satisfying the condition HPBW-S ≤ 18.8° ≤ HPBW-L.

(*θ_S_*, *θ_L_*)	HPBW-S (°)	HPBW-L (°)	*R_BW_* (Dimensionless)	Amplitude Gain (dB)
(50°, 50°)	18.8	18.8	1.00	9.0
(40°, 70°)	17.1	33.3	1.95	7.0
(50°, 70°)	15.9	30.7	1.93	8.1
(50°, 80°)	15.3	34.3	2.24	6.1
(60°, 70°)	18.1	22.1	1.22	7.8

**Table 2:** Comparison of experimental results for conical transducer cavities with different angles. HPBW denotes the half-power beamwidth of acoustic directivity in the plane of the flow direction in the pipe.

Angle	Experiment HPBW (°)	Experiment Amplitude Gain (dB)
(40°, 40°)	22.8	5.6
(50°, 50°)	19.1	7.8
(60°, 60°)	23.6	5.4
(70°, 70°)	35.5	3.1
(80°, 80°)	56.5	1.5
(40°, 70°)	31.9	6.7
(50°, 70°)	29.1	7.4
(50°, 80°)	36.8	5.7
(60°, 70°)	33.8	4.1

## Data Availability

Data are contained within the article.
